# Watch-and-wait approach versus adjuvant treatment after radical awake resection in selected adult-type grade 3 gliomas, *isocitrate dehydrogenase* mutant: A case-matched cohort

**DOI:** 10.1093/noajnl/vdae189

**Published:** 2024-11-18

**Authors:** Angela Elia, Alexandre Roux, Bénédicte Trancart, Alessandro Moiraghi, Maimiti Seneca, Edouard Dezamis, Pascale Varlet, Fabrice Chretien, Catherine Oppenheim, Marc Zanello, Johan Pallud

**Affiliations:** Faculté de Médecine, Université Paris Cité, Paris, France; Faculté de Médecine, Université Paris Cité, Paris, France; Faculté de Médecine, Université Paris Cité, Paris, France; Department of Neurosurgery, GHU Paris Psychiatrie et Neurosciences - Sainte-Anne Hospital, Paris, France; Inserm, U1266, IMA-Brain, Centre de Psychiatrie et Neurosciences, Paris, France; Faculté de Médecine, Université Paris Cité, Paris, France; Department of Neurosurgery, GHU Paris Psychiatrie et Neurosciences - Sainte-Anne Hospital, Paris, France; Faculté de Médecine, Université Paris Cité, Paris, France; Faculté de Médecine, Université Paris Cité, Paris, France; Department of Neurosurgery, GHU Paris Psychiatrie et Neurosciences - Sainte-Anne Hospital, Paris, France; Department of Neuropathology, GHU Paris Psychiatrie et Neurosciences - Sainte-Anne Hospital, Paris, France; Inserm, U1266, IMA-Brain, Centre de Psychiatrie et Neurosciences, Paris, France; Inserm, U1266, IMA-Brain, Centre de Psychiatrie et Neurosciences, Paris, France; Faculté de Médecine, Université Paris Cité, Paris, France; Department of Neuroradiology, GHU Paris Psychiatrie et Neurosciences - Sainte-Anne Hospital, Paris, France; Faculté de Médecine, Université Paris Cité, Paris, France; Inserm, U1266, IMA-Brain, Centre de Psychiatrie et Neurosciences, Paris, France; Faculté de Médecine, Université Paris Cité, Paris, France; Faculté de Médecine, Université Paris Cité, Paris, France; Department of Neurosurgery, GHU Paris Psychiatrie et Neurosciences - Sainte-Anne Hospital, Paris, France

**Keywords:** awake surgery, grade 3 glioma, radiotherapy, survival, watch-and-wait

## Abstract

**Background:**

Following large resection, proposing a watch-and-wait strategy in selected grade 3 glioma, *isocitrate dehydrogenase* (*IDH)*-mutant patients is an emerging practice. We compared the watch-and-wait approach to the standard postoperative adjuvant oncological treatment for grade 3 gliomas, *IDH*-mutant.

**Methods:**

Observational, retrospective, single-institution cohort (2011–2023) of 106 consecutive adult patients harboring supratentorial grade 3 gliomas, *IDH*-mutant treated by maximal awake resection and who received a watch-and-wait approach (surgery group) or an adjuvant oncological treatment (oncological group) postoperatively. Case-matched analysis (1:1) criteria between the surgery group and oncological group: extent of resection, tumor volume, Karnofsky Performance Status (KPS) score, tumor location and size, and age.

**Results:**

Patients of the surgery group (*n* = 26) had significantly better KPS scores, less preoperative neurological and/or neurocognitive deficits, less hyperperfusion, less corpus callosum infiltration, smaller tumor volume, higher rate of total resection, and smaller residual tumor than patients of the oncological group (*n* = 80). The 5-year progression-free survival (66.2 vs. 77.9 months, *P* = .713) and the 5-year overall survival (88.9 vs. 83.9 months, *P* = .291) did not differ between surgery and oncological groups. In the whole series, a preoperative KPS score >70, a total resection, and the oligodendroglioma subtype were independent predictors of longer progression-free survival and overall survival. After case matching, no difference in survival was observed between watch-and-wait and oncological treatment both in astrocytomas (*n* = 14 per group) and oligodendrogliomas (*n* = 12 per group).

**Conclusions:**

Watch-and-wait following radical resection appears to be feasible in highly selected grade 3 gliomas, *IDH*-mutant patients without impairing survival both in astrocytoma and in oligodendroglioma subgroups.

## Lay Summary

Gliomas are a type of brain tumor. Gliomas are graded between grades 2 and 4 by pathologists, with higher grades being more aggressive tumors. Some doctors recommend additional treatments like chemotherapy and radiation right after surgery, while others may not suggest any more immediate treatment for grade 3 gliomas. The authors of this study wanted to see which approach is better. To do this they reviewed the medical records of 106 patients with grade 3 gliomas some of whom received chemotherapy and/or radiation immediately after surgery and others who did not receive any immediate treatment after surgery. Their results showed that there was no difference in how long patients lived, regardless of the treatment they received immediately after surgery.

**Key Points**

- In grade 3 gliomas, *IDH*-mutant, the watch-and-wait approach after radical awake resection was not associated with earlier tumor progression.
- A watch-and-wait approach after radical resection is feasible in grade 3 gliomas, *IDH*-mutant without impairing survival.

**Importance of the Study**

In this single-center cohort of 106 consecutive highly selected adult patients who underwent maximal awake resection for a newly diagnosed supratentorial grade 3 glioma, *IDH*-mutant, we show that: (1) preoperative Karnofsky Performance Status (KPS) score >70, total resection, and oligodendroglioma subtype were independent predictors of longer survivals; (2) patients selected for a watch-and-wait approach following surgery had significantly better KPS score, less preoperative neurological/neurocognitive deficit, less hyperperfusion, less corpus callosum infiltration, smaller tumor volume, and higher rate of total resection than patients received standard oncological treatment; (3) the watch-and-wait approach following surgery was not associated with early tumor progression and did not negatively impact progression-free-survival and OS both in astrocytoma and in oligodendroglioma subgroups; and (4) the 5-year PFS and the 5-year OS did not significantly differ between watch-and-wait approach and standard oncological treatment. A watch-and-wait approach following radical resection appears to be feasible in highly selected grade 3 gliomas, *IDH*-mutant without impairing survival.

Adult-type diffuse gliomas represent a heterogeneous tumor group. In the 2021 World Health Organization (WHO) Classification of Central Nervous System (CNS) tumors, diffuse gliomas are classified and graded based on the integrated histo-molecular diagnosis.^1^ Among gliomas *isocitrate dehydrogenase (IDH)* 1/2-mutant, the distinction between grade 2 and grade 3 remains governed by morphologic features (including mitotic activity). Although the clinical validity of distinguishing between grade 2 and 3 gliomas, *IDH*-mutant has been largely questioned,^2–5^ the oncological treatment differs with the grade of malignancy. Current practice recommendations for grade 3 gliomas, *IDH*-mutant are a maximal safe surgical resection whenever feasible followed by radiotherapy (54 to 60 Gy in 1.8 to 2.0 Gy daily fractions, started 3 to 6 weeks after surgery) and chemotherapy (adjuvant PCV or adjuvant Temozolomide for oligodendroglioma, *IDH*-mutant and 1p/19q-codeleted, adjuvant Temozolomide for astrocytoma, *IDH*-mutant).^6–10^

Since radiotherapy is associated with long-term toxicities, including neurocognitive impairment,^11–16^ their occurrence in patients harboring a grade 3 glioma, *IDH*-mutant with a long-expected survival is at high risk of impacting the health-related quality of life.^17^ This questions the possibility of postponing oncological treatments in such patients with favorable clinical and histo-molecular profiles. Society for Neuro-Oncology, American Society of Clinical Oncology, and American Society for Radiation Oncology (ASTRO) guidelines state that early radiotherapy followed by chemotherapy is strongly recommended for all grade 3 glioma *IDH*-mutant patients.^7,8,10^ European Association of Neuro-Oncology (EANO) guidelines suggest that a watch-and-wait approach can be considered in grade 3 oligodendroglioma, *IDH*-mutant and 1p/19q-codeleted, in younger patients ≤40 years, following total resection, in the absence of neurological deficit, and without homozygous *CDKN2A/B* deletion.^6^ Revisiting the treatment paradigm in *IDH*-mutant grade 3 gliomas is therefore wise and necessary.

In the present study, we reviewed the oncological management of newly diagnosed supratentorial grade 3 gliomas, *IDH*-mutant in a homogeneous single-institution cohort of adult patients. In selected patients, we assessed the frequency, evolution over time, feasibility, and survival impact of a watch-and-wait approach following maximal safe function-based awake resection avoiding postoperative adjuvant oncological treatment. We compared this watch-and-wait approach to the standard early postoperative adjuvant oncological treatment using case matching for both grade 3 oligodendrogliomas, *IDH*-mutant and 1p/19q-codeleted and astrocytomas, *IDH*-mutant.

## Materials and Methods

### Study Design and Setting

We performed an observational, retrospective, and consecutive cohort study in a single tertiary referral neurosurgical center between April 2011 and April 2023.

### Ethics Statement

The study protocol was approved by the local institutional review board (Protocol number A01933-48). The manuscript was written according to the Strengthening the Reporting of Observational Studies in Epidemiology (STROBE) checklist.^18^

### Participants

Inclusion criteria were: (1) age ≥18 years; (2) no previous oncological treatment; (3) diagnosis of grade 3 astrocytoma, *IDH*-mutant or of grade 3 oligodendroglioma, *IDH*-mutant and 1p/19q-codeleted according to the 2021 WHO classification^1^; (4) patients operated on using the same procedure of maximal safe function-based awake resection; and (5) available imaging and clinical follow-up data.

### Histopathological Central Review

All tumors were classified according to the WHO 2021 classification with a histo-molecular reassessment by a certified neuropathologist for all cases diagnosed prior to September 2021.^1^ The screening for *IDH1R132H* mutation was carried out using immunohistochemistry. For cases with a negative IDH1R132H immunohistochemistry, a sequencing of *IDH1/2* mutations had additionally been performed to identify minor *IDH1/2* mutations. A sequencing of 1p19q-codeletion had been systematically performed.

A diagnosis of oligodendroglioma, *IDH*-mutant, and 1p/19q-codeleted CNS WHO grade 3 was made in cases with increased mitotic activity (≥6 mitoses per 10 consecutive 0.24 mm^2^ areas) with or without neoangiogenesis and with or without necrosis. A diagnosis of astrocytoma, *IDH*-mutant, CNS WHO grade 3 was made in cases with increased mitotic activity (≥1 mitosis within a biopsy sample, ≥2 mitoses per 10 consecutive 0.24 mm^2^ areas within a surgical resection sample) without neoangiogenesis and without necrosis.

### Data Collection

Data were systematically gathered from medical records using a protocol designed for the study. Clinical characteristics at the time of histo-molecular diagnosis were: age, sex, Karnofsky performance status (KPS) score, signs of raised intracranial pressure at diagnosis, focal neurological deficit and/or neurocognitive deficit at diagnosis, epileptic seizures at diagnosis. Imaging characteristics on preoperative MRI were: main tumor location and side (ie, where the tumor was predominantly located for multiple lobar involvements), presence and pattern of contrast enhancement as previously described,^19^ tumor volume (quantified by segmentation of all visible signal abnormalities on fluid-attenuated inversion recovery (FLAIR) sequence). Treatment-related characteristics were: the extent of surgical resection and residual tumor quantified on early (<48 hours) postoperative FLAIR sequence (total resection when no residual abnormality was present, partial resection in the remaining cases), adjuvant oncological treatment (none, radiotherapy with dose, chemotherapy with molecule, dose, and duration), tumor progression, oncological treatment at progression, and death.

Progression-free survival (PFS) was measured from the date of surgery to the date of tumor progression requiring new oncological treatment according to advanced MRI and followed the criteria proposed by the Response Assessment in Neuro-Oncology working group.^20–22^ Overall survival (OS) was measured from the date of diagnosis to the date of death from any cause.

### Surgical Procedure

A reproducible “asleep-awake-asleep” surgical protocol was used by the same senior neurosurgeon to achieve maximal safe function-based resection, as previously detailed.^23–28^ The intraoperative functional brain mapping was performed by cortical and subcortical direct electrical stimulations during the awake phase of surgery (bipolar electrode, 5-mm distance between tips, frequency of 60 Hz; biphasic current, phase duration of 1 milliseconds; stimulation duration of 4 seconds; Osiris NeuroStimulator, Inomed). The current intensity was calibrated by stimulating the sensory/motor area with a progressive amplitude increase (baseline 1 mA, 0.5 mA increments) until reproducible positive responses were elicited. The same current intensity was used for further cortical and subcortical functional mapping and resection. A senior speech therapist checked intraoperatively neurological and neurocognitive functions using defined tasks throughout the awake phase.^24,29,30^ The glioma was progressively removed, guided by subcortical functional mapping while the awake patient performed the required tasks. Resections were stopped when eloquent subcortical structures around the surgical cavity were identified or until the patient became fatigued with testing.^26,28^

### Statistical Analyses

To assess the survival impact of postoperative oncological management in grade 3 gliomas, *IDH*-mutant, we performed a case-matched analysis (1:1 matching) by comparing patients who received a watch-and-wait approach following maximal-safe function-based awake resection (surgery group) with patients who received a standard adjuvant oncological treatment following surgery (oncological group). Each patient in the surgery group was individually matched with a patient in the oncological group according to the following 6 hierarchical criteria: (1) extent of resection (total vs. partial); (2) preoperative FLAIR volume (<45 vs. ≥45 cc); (3) preoperative KPS score (≤70 vs. >70); (4) tumor location (same lobe); (5) glioma side; and (6) age (≤40 vs. >40 years). If any control matched all criteria, tolerances for age and for tumor location (frontal with parietal and/or insular) were applied. **Supplementary Table 1** shows the characteristics of all matched pairs.

Descriptive statistics were reported as the mean ± standard deviation for continuous variables and as a percentage for categorical variables. Univariate analyses were carried out using the chi-square or Fisher’s exact tests for comparing categorical variables, and the unpaired t-test or Mann-Whitney rank sum test for continuous variables, as appropriate. Unadjusted survival curves for OS and PFS were plotted by the Kaplan–Meier method, using log-rank tests to assess the significance for group comparison. We then constructed Cox proportional hazards regression models on the whole series using a backward stepwise approach, entering the predictors previously associated with mortality and progression in univariate analysis at the *P* < .1 level. A *P*-value <.050 was considered significant.

Analyses were performed using JMP software (Version 18.0.2; SAS Institute Inc.).

## Results

### Study Population

A total of 106 patients were included (60 grade 3 astrocytomas, *IDH*-mutant, 46 grade 3 oligodendrogliomas, *IDH*-mutant, and 1p19q-codeleted). No patient was excluded during the study period. The patients’ main characteristics are detailed in **Table 1**. The 106 patients (64 males) had a mean age of 42 ± 14 years (range, 18–74). Among them, 26 patients received a watch-and-wait approach following maximal-safe function-based awake resection (surgery group) while 80 patients received an early standard adjuvant oncological treatment following surgery (oncological group). Patients of the surgery group had significantly better KPS scores (*P* = .047), less preoperative neurological focal deficit (*P* = .024), less preoperative neurocognitive deficit (*P* = .013), less hyperperfusion (*P* = .001), less corpus callosum infiltration (*P* = .002), smaller tumor volume (*P* = .003), higher resection rates (total resection rate: 80.7 Vs 35.0%, *P* < .001; mean extent of resection: 96.5% ± 9.8% vs. 86.3% ± 18.4%, *P* < .001), and smaller residual tumor (mean extent of resection: 1.5 ± 4.9 vs. 9.3 ± 14.8 cm^3^, *P* < .001) than patients of the oncological group.

**Table 1.** Main Characteristics of the Study Sample (*n* = 106)

	Whole series (*n* = 106)		Surgery group (*n* = 26)		Oncological group (*n* = 80)		*P*-value
Parameters	*n*	%	*n*	%	*n*	%	
Sex							.889
Male	64	60.38	16	61.54	48	60.00	
Female	42	39.62	10	38.46	32	40.00	
**Age, years (median, mean ± SD, range)**			40.5, 41.61 ±15.66, 21–72		42, 42.60 ±12.94, 16–74		.537
≤40	50	47.17	13	50.00	37	46.25	.739
>40	56	52.83	13	50.00	43	52.83	
**Handedness**							.597
Right	97	91.51	25	96.15	72	90.00	
Left	8	7.55	1	3.85	7	8.75	
Ambi	1	0.94	0	0.00	1	1.25	
**Preoperative KPS score (median, mean ± SD, range)**	100, 96 ±9.21, 50–100		100, 98.46 ±4.64, 80–100		100, 94.62 ±10.30, 50–100		.047
≤70	2	1.89	0	0.00	2	2.50	.415
>70	104	98.11	26	100.00	78	97.50	
**Epileptic seizure onset**							.644
Yes	81	76.42	19	73.08	62	77.50	
No	25	23.58	7	26.92	18	22.50	
Preoperative epileptic seizure control *							.897
No	8	9.76	2	10.53	6	9.52	
Yes	74	90.24	17	89.47	57	90.48	
Preoperative signs of raised intracranial pressure **							.982
No	102	96.23	25	96.15	77	96.25	
Yes	4	3.77	1	3.85	3	3.75	
Preoperative neurological focal deficit ***							.024
No	86	81.13	25	96.15	61	76.25	
Yes	20	18.87	1	3.85	19	23.75	
**Preoperative neurocognitive deficit**							.013
No	25	23.58	11	42.31	14	17.50	
Yes	81	76.42	15	57.69	66	82.50	
Tumor side							.645
Right	44	41.51	10	38.46	34	42.50	
Left	60	56.60	16	61.54	44	55.00	
Bilateral	2	1.89	0	0.00	2	2.50	
**Main tumor location**							.551
Frontal	53	50.00	12	46.15	41	51.25	
Insular	19	17.92	7	26.92	12	15.00	
Temporal	17	16.04	4	15.38	13	16.25	
Parietal	17	16.04	3	11.54	14	17.50	
**Contrast enhancement**							.103
Yes	43	40.57	7	26.92	36	45.00	
No	63	59.43	19	73.08	44	55.00	
**Type of contrast enhancement**							.253
None	62	59.43	19	73.08	44	55.00	
Faint and patchy	21	19.81	5	19.23	16	20.00	
Nodular-like	19	17.92	2	7.69	17	21.25	
Ring-like	3	2.83	0	0.00	3	2.83	
**Hyperperfusion**							.001
Yes	50	47.17	5	19.23	45	56.25	
No	55	51.89	20	76.92	35	43.75	
Missing	1	0.94	1	3.85	0	0.00	
**Mass effect on the midline**							.514
Yes	21	19.81	4	15.38	17	21.25	
No	85	80.19	22	84.62	63	78.75	
**Callosum body infiltration**							.002
Yes	15	14.15	0	0.00	15	18.75	
No	91	85.85	26	100.00	65	81.25	
**Ventricular contact**							.068
Yes	49	46.23	8	30.77	41	51.25	
No	57	53.77	18	69.23	39	48.75	
**Tumor volume at surgery, cm3 (median, mean ± SD, range)**	44.20, 60.68± 48.31, 3.5 – 207.1		28.65, 32.33±23.65, 3.5 – 108.3		59.65, 69.89 ±50.74, 6.3 – 207.1		<.001
<45	55	51.89	20	76.92	35	43.75	.003
≥45	51	48.11	6	23.08	45	56.25	
**Extension of resection, % (median, mean ± SD, range)**	96.5, 88.76±17.25, 4.5-100		100, 96.48±9.84, 54.9-100		93.0, 86.25±18.41, 4.5-100		<.001
Partial	67	53.78	5	19.23	52	65.00	<.001
Total	49	46.22	21	80.77	28	35.00	
**Residual tumor, cm3 (median, mean ± SD, range)**	1.45, 7.38±15.52, 0-43.60		0.0, 1.49±4.87, 0-20.4		4.04, 9.29±14.84, 0-43.60		<.001
**Histo-molecular diagnosis**							.744
Astrocytoma, IDH-mutant CNS WHO grade 3	60	56.60	14	53.85	46	57.50	
Oligodendroglioma, IDH-mutant CNS WHO grade 3	46	43.40	12	46.15	34	42.50	
**Reason for oligodendroglioma, IDH-mutant CNS WHO grade 3**							.053
Mitosis cell count ≥6 only	33	71.74	11	91.67	22	64.71	
Neoangiogenesis and/or necrosis	13	28.26	1	08.33	12	35.29	
**Tumor progression**							.514
No	85	80.19	22	84.62	63	78.75	
Yes	21	19.81	4	15.38	17	21.25	
**Treatment at first progression**							
Surgery	4	19.05	2	50.00	2	11.76	.079
Radiotherapy	4	19.05	1	25.00	3	17.65	.736
Chemotherapy	13	61.90	1	25.00	12	70.59	.091

CNS, central nervous system; IDH, isocitrate dehydrogenase; KPS, Karnofsky Performance Status; SD, standard deviation; WHO, World Health Organization.

*Diagnosed on a clinical basis with or without confirmation by an electroencephalogram.

**ie, headache, nausea/vomiting, coma, diplopia, visual blur, papilledema.

*** ie, motor deficit, sensitive deficit, language disorders, visual disturbances, and frontal syndrome.

**Figure 1.**
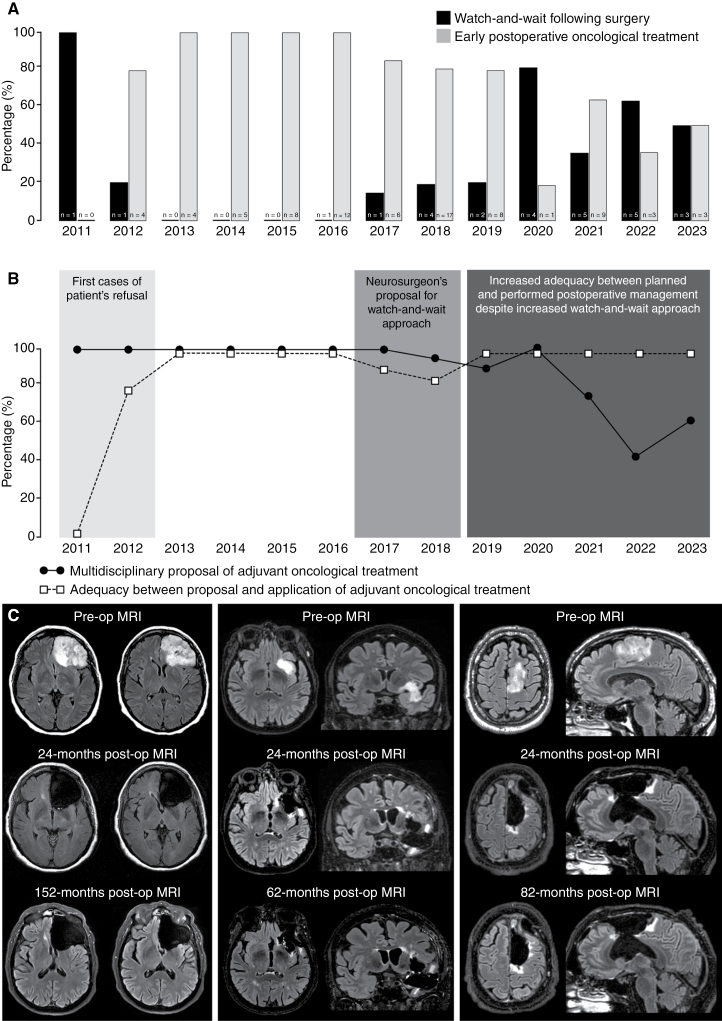
Comparison over time between planned and performed oncological management following maximal safe function-based resection of grade 3 gliomas, *IDH*-mutant. (A) Evolution over time of the watch-and-wait approach versus early adjuvant oncological treatment postoperatively. (B) Evolution over time between the planned and the performed oncological management postoperatively. Before 2017, the early adjuvant treatment was decided in multidisciplinary meetings and applied in all cases, except in rare cases of patient refusal. Since 2017, a watch-and-wait approach has increasingly been proposed by the referring neurosurgeons and accepted by the patients despite the decision of early adjuvant treatment in multidisciplinary meetings. Since 2019, the watch-and-wait approach has increasingly proposed adequacy between the postoperative management decided in multidisciplinary meetings and accepted by the patients. (C) Illustrative cases of patients who received a watch-and-wait approach following maximal-safe function-based awake resection. Left panel: preoperative, 24-month postoperative, and 152-month postoperative MRI of patient n°1 of the surgery group (Supplementary Table 2). Middle panel: preoperative, 24-month postoperative, and 62-month postoperative MRI of patient *n*°6 of the surgery group (Supplementary Table 2). Right panel: preoperative, 24-month postoperative, and 82-month postoperative MRI of patient *n*°3 of the surgery group (Supplementary Table 2).

Characteristics of the patients of the surgery group are detailed in **Supplementary Table 2**. The reasons for no postoperative adjuvant oncological treatment in these patients include a multidisciplinary decision of watch-and-wait approach in 10 cases (38.5%) and a multidisciplinary decision of adjuvant oncological treatment not applied in 16 cases: following referent neurosurgeon’s proposal in 11 cases (42.3%), following patient’s decision in 4 cases (15.4%), and due to poor patient’s condition in one case (3.8%). The evolution over time of the watch-and-wait approach following maximal-safe function-based resection under awake conditions for a grade 3 glioma, *IDH*-mutant is illustrated in **Figure 1**.

Patients in the oncological group received both radiotherapy (mean dose, 59.4 ± 1.4 Gy; range 50.4–60) and chemotherapy (PCV in 28 cases, temozolomide in 48 cases) in 75 cases; they received radiotherapy only in 4 cases, and PCV chemotherapy only in one case. Postoperative adjuvant oncological treatment was started at a mean of 2.5 ± 1.3 months (range 1-9) following surgery.

### Progression-Free Survival and Overall Survival Analyses

During the follow-up period (median, 55.2 months; range, 12–152), 21 patients (19.8%) experienced disease progression requiring further oncological treatment, and 14 patients (13.2%) died.

**Figure 2.**
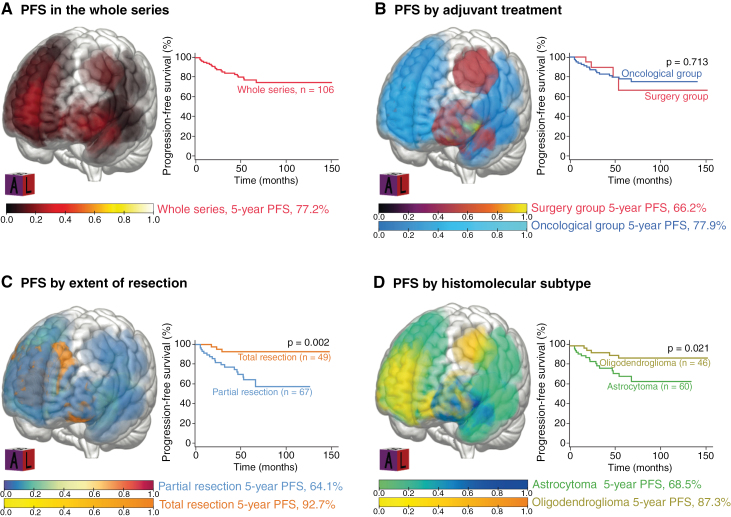
Progression-free survival of grade 3 gliomas, *IDH*-mutant (*n* = 106) in the whole series (A), by adjuvant oncological treatment (B), by extent of resection (C), and by histo-molecular subtype (D).

A tumor progression occurred in 4/26 patients (14.6%, median 36 months, range 18.0–54.0) of the surgery group and in 17/80 patients (21.3%, median 22 months, range 5.5–68.0) of the oncological group. No progression was observed in the first 18 postoperative months in the surgery group. Progression-free survivals are illustrated in **Figure 2**. The median PFS was not reached in the whole series. The 5-year PFS was 77.2% in the whole series, without a significant difference between the surgery group (66.2%) and the oncological group (77.9%, *P* = .713). In multivariable analyses (Table 2), a preoperative KPS score ≤70 compared to >70 (adjusted hazard ratio [aHR] 19.07 [95% confidence interval (CI) 2.07-175.45], *P* = .009), a residual tumor (aHR 1.03 per cm^3^ of residual [95% CI 1.00–1.05], *P* = .028), and the astrocytoma subtype compared to the oligodendroglioma subtype (aHR 3.59 [95% CI 1.13–11.39], *P* = .029) were associated with a shorter PFS. The watch-and-wait approach following surgery was not a predictor for PFS. All patients with a progressive disease received second-line oncological treatments detailed in Table 1.

**Table 2.** Univariate and Multivariate Predictors of Progression-Free Survival in the Whole Series (*n* = 106)

Parameters		uHR	CI95%	*P*-value	aHR	CI95%	*P*-value
Sex	Female	1 (ref)					
	Male	1.37	0.55–3.41	.487			
Age, year	≤40	1 (ref)					
	>40	0.97	0.41–2.30	.957			
Preoperative KPS score	>70	1 (ref)			1 (ref)		
	≤70	73.00	9.99–533.14	**<.001**	19.07	2.06–176.45	**.009**
Corpus callosum involvement	No	1 (ref)					
	Yes	1.81	0.66–4.94	.246			
Glioma location	Frontal	1 (ref)					
	Temporal	1.22	0.41–3.62	.723			
	Insular	1.17	0.39–3.49	.774			
	Parietal	0.60	0.14–2.58	.496			
Glioma side location	Left	1 (ref)					
	Right	1.23	0.52–2.91	.627			
Tumor volume, cm^3^	<45	1 (ref)					
	≥45	2.52	0.97–6.49	.056			
Hyperperfusion	No	1 (ref)					
	Yes	1.92	0.78–4.78	.367			
	Missing	N/A					
Contrast enhancement	None	1 (ref)					
	Faint and patchy	0.70	0.19–2.48	.580			
	Nodular-like	1.14	0.40–3.27	.796			
	Ring-like	1.75	0.22–13.49	.592			
Residual tumor, cm^3^	Per unit	1.02	1.00–1.04	**.017**	1.03	1.00–1.05	**.028**
Histo-molecular diagnosis	Oligodendroglioma	1 (ref)			1 (ref)		
	Astrocytoma	3.15	1.15–8.62	**.025**	3.59	1.13–11.39	**.029**
Oncological treatment	Surgery only	1 (ref)					
	Surgery + RT/CHT	1.02	0.34–3.06	.969			

aHR, adjusted hazard ratio; CI, confidence interval; CHT, chemotherapy; KPS, Karnofsky Performance Status; RT, radiotherapy; uHR, unadjusted hazard ratio.

Unadjusted hazard ratios by log-rank tests and adjusted hazard ratios by Cox proportional hazards model. *P*-values in bold are significant.

**Figure 3.**
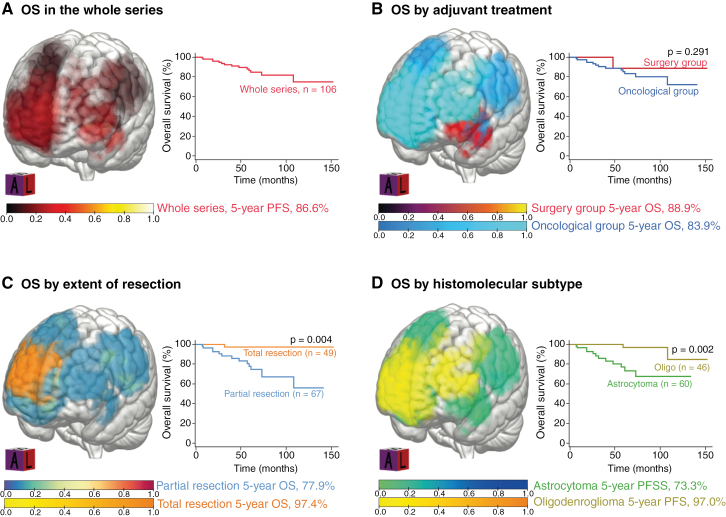
Overall Survival of grade 3 gliomas, *IDH*-mutant (*n* = 106) in the whole series (A), by adjuvant oncological treatment (B), by extent of resection (C), and by histo-molecular subtype (D).

Overall survivals are illustrated in **Figure 3**. The median OS was not reached in the whole series. The 5-year OS was 86.5% in the whole series, without a significant difference between the surgery group (88.9%) and the oncological group (83.9%, *P* = .291). In multivariable analyses (**Table 3),** a preoperative KPS score ≤70 compared to >70 (aHR 10.64 [95% CI 1.66–68.29], *P* = .013), a residual tumor (aHR 1.02 per cm^3^ of residual [95% CI 1.00–1.04], *P* = .023), and the astrocytoma subtype compared to the oligodendroglioma subtype (aHR 5.26 [95% CI 1.12–24.66], *P* = .035) were associated with a shorter OS. The watch-and-wait approach following surgery was not a predictor for OS.

**Table 3.** Univariate and Multivariate Predictors of Overall Survival in the Whole Series (*n* = 106)

		uHR	CI 95%	*P*-value	aHR	CI 95%	*P*-value
Parameters							
Sex	Female	1 (ref)					
	Male	2.47	0.68–10.32	.166			
Age, year	≤40	1 (ref)					
	> 40	1.58	0.53–4.73	.409			
Preoperative KPS score	> 70	1 (ref)			1 (ref)		
	≤70	35.71	5.90–216.02	**<.0001**	10.64	1.66–68.29	**.013**
Corpus callosum involvement	No	1 (ref)					
	Yes	1.92	0.59–6.14	.272			
Glioma location	Frontal	1 (ref)					
	Temporal	1.37	0.38–4.95	.625			
	Insular	1.78	0.48–6.53	.381			
	Parietal	1.08	0.23–4.87	.921			
Glioma side location	Left	1 (ref)					
	Right	1.03	0.35–3.04	.948			
Tumor volume, cc	<45	1 (ref)					
	≥45	2.10	0.65–6.75	.211			
Hyperperfusion	No	1 (ref)					
	Yes	2.19	0.68–7.04	.419			
	Missing	N/A					
Contrast enhancement	None	1 (ref)					
	Faint and patchy	0.33	0.04–2.65	.297			
	Nodular-like	1.29	0.38–4.31	.683			
	Ring-like	2.11	0.26–17.19	.486			
Residual tumor, cm^3^	Per unit	1.03	1.01–1.05	**.003**	1.02	1.00–1.04	**.023**
Histo-molecular diagnosis	Oligodendroglioma	1 (ref)			1 (ref)		
	Astrocytoma	6.89	1.53–31.02	**.012**	5.26	1.12–24.66	**.035**
Oncological treatment	Surgery only	1 (ref)					
	Surgery + Radio-chemotherapy	2.73	0.35–21.03	.335			

aHR, adjusted hazard ratio; CI, confidence interval; CHT, chemotherapy; KPS, Karnofsky Performance Status; RT, Radiotherapy; uHR, unadjusted hazard ratio.

Unadjusted hazard ratios by log-rank tests and adjusted hazard ratios by Cox proportional hazards model. *P*-values in bold are significant.

### Case-Matched Analysis in Grade 3 Oligodendroglioma, *IDH*-Mutant and 1p19q-Codeleted

The 12 patients with a grade 3 oligodendroglioma, *IDH*-mutant, and 1p19q-codeleted of the surgery group were matched with 12 patients with a grade 3 oligodendroglioma, *IDH*-mutant and 1p19q-codeleted of the oncological group. The case-matching (*n* = 12 in both groups) is detailed in **Supplementary Table 1**. No significant difference among matching criteria was observed in the case–control sampling as detailed in **Supplementary Figure 1**.

The estimated 5-year PFS did not significantly differ between the surgery group (85.3%) and the oncological group (100%, *P* = .083). After case matching, no significant difference in PFS was observed between the 2 groups (median not reached, *P* = .083; **Supplementary Figure 2**). The estimated 5-year OS did not significantly differ between the surgery group (100%) and the oncological group (100%, *P* = 1.0). After case matching, no significant difference in OS was observed in the 2 groups (median not reached, *P* = .479; **Supplementary Figure 2****).**

### Case-Matched Analysis in Grade 3 Astrocytoma, *IDH*-Mutant

The 14 patients with a grade 3 astrocytoma, *IDH*-mutant of the surgery group were matched with 14 patients with a grade 3 astrocytoma, *IDH*-mutant of the oncological group. The case-matching (*n* = 14 in both groups) is detailed in **Supplementary Table 1**. No significant difference among matching criteria was observed in the case–control sampling as detailed in **Supplementary Figure 1**.

The estimated 5-year PFS did not significantly differ between the surgery group (100%) and the oncological group (100%, *P* = 1.0). After case matching, no significant difference in PFS was observed between the 2 groups (median 48.0 months in the surgery group, not reached in the oncological group, *P* = .178; **Supplementary Figure 2**). The estimated 5-year OS did not significantly differ between the surgery group (100%) and the oncological group (100%, *P* = 1.0). After case matching, no significant difference in OS was observed in the 2 groups (median not reached, *P* = .479; **Supplementary Figure 2**).

## Discussion

### Key Results

In this single-center cohort of 106 consecutive adult patients who underwent maximal safe function-based awake resection for a newly diagnosed supratentorial grade 3 glioma, *IDH*-mutant, we show that: (1) a preoperative KPS score >70, a total resection, and the oligodendroglioma subtype were independent predictors of longer PFS and OS; (2) patients of the surgery group had significantly better KPS score, less preoperative neurological focal deficit and neurocognitive deficit, less hyperperfusion, less corpus callosum infiltration, smaller tumor volume, higher rate of total resection, and smaller residual tumor than patients of the oncological group; (3) the wait-and-watch approach following maximal safe function-based awake resection was not associated with early tumor progression and did not negatively impact PFS and OS both in astrocytoma and in oligodendroglioma subgroups; (4) the 5-year PFS and the 5-year OS did not significantly differ between surgery and oncological groups.

### Interpretation

In the present study, we assessed the impact of proposing a watch-and-wait approach rather than the standard early adjuvant radio-chemotherapy following maximal safe function-based awake resection of supratentorial grade 3 gliomas, *IDH*-mutant in selected low-risk patients. Current practical recommendations following surgery are radiotherapy and adjuvant chemotherapy.^6–10^ Although these standards of care are based on robust clinical trials completed before the current WHO classification, the question of their optimal timing remains uncertain. Particularly, it is unclear whether the benefits of early oncological treatment, including radiotherapy, outweigh potential side-effects of acute and long-term toxicity, and which clinical or molecular factors optimally identify patients that are the best candidates for early oncological treatment or wait and watch approach.^31^ Postponing historical oncological treatments is a topical question with the arrival of *IDH1/2* inhibitors for the treatment of gliomas, *IDH*-mutant.

However, EANO guidelines have suggested in 2021 that a watch-and-wait approach can be considered in grade 3 oligodendroglioma, *IDH*-mutant, and 1p/19q-codeleted patients with a favorable profile^6^ but reports of such a conservative approach are rare. A single-center retrospective study, including 44 patients with a diffuse low-grade glioma according to the 2016 WHO classification (IDH-mutant in 88% of cases, no 1p19q codeletion in 59% of cases) encompassing foci of high-grade features (grade 3 or 4), postponed adjuvant oncological treatment after subtotal or total resection.^32^ They reported a tumor progression requiring further oncological treatment in 75% of patients, after a median time of 3.4 years since initial surgery, suggesting the feasibility of an initial conservative approach without affecting survival in such diffuse low-grade gliomas.^32^ Another retrospective study, including 6 patients with diffuse low-grade glioma according to various WHO classifications with foci of anaplasia, postponed adjuvant oncological treatment after maximal safe resection.^33^ They reported no recurrence and adjuvant treatment at a mean of 58 months-follow-up, suggesting that following maximal safe resection, the presence of small malignant foci embedded within a grade 2 glioma may be followed up without immediate adjuvant therapy.^33^ If these 2 studies reported diffuse low-grade gliomas with foci of anaplasia, possibly to justify the applied watch-and-wait approach, the reported cases were, actually, grade 3 gliomas since the WHO classification of the tumors of the CNS does not recognize intermediate grading. These results are striking since the proposal of maximal resection plus the watch-and-wait approach was applied to actual malignant gliomas with satisfying reported outcomes.^32,33^ A retrospective study, including 201 patients with a grade 2 (*n* = 131) or 3 (*n* = 70) oligodendroglioma, *IDH*-mutant and 1p/19q-codeleted according to the 2021 WHO classification, assessed the impact of early postoperative treatment and watch-and-wait approach.^34^ They reported that the early postoperative oncological treatment and the grade of malignancy were not associated with PFS.^34^

Based on these results and on our own clinical experience, we have already proposed a watch-and-wait approach following maximal resection in selected cases of high-grade diffuse gliomas.^35^ In the present series, we reported our preliminary experience in newly diagnosed supratentorial grade 3 glioma, *IDH*-mutant according to the 2021 WHO classification treated with maximal safe function-based awake resection followed by a watch-and-wait approach and no early adjuvant therapy. Such a watch-and-wait approach has been proposed in selected grade 3 glioma, *IDH*-mutant patients with favorable clinical, imaging, and histo-molecular profiles and with a high rate of tumor resection. We reported non-inferior preliminary outcomes, the PFS and the OS being similar between the surgery and the oncological groups. The present results indirectly suggest that the maximal safe function-based resection using intraoperative brain functional mapping under awake conditions per se has a strong and positive impact on survival of grade 3 gliomas, *IDH*-mutant. This supports the possibility of a watch-and-wait approach following radical resection in selected grade 3 astrocytoma and oligodendroglioma patients. Learning from the experience, we are increasingly proposing such a conservative approach in selected patients with a favorable profile, including a large function-based resection, beyond guidelines from grade 3 gliomas, *IDH*-mutant, as illustrated by the increase of the multidisciplinary decision of a watch-and-wait approach over time. In addition, the present results question the relevance of a mitosis count cutoff, which is a subjective measure, when attempting to grade a glioma, *IDH*-mutant. Previous studies have demonstrated that glioma aggressiveness estimated by the tumor growth rate on imaging increased in cases with a mitosis cell count ≥4 per 10 consecutive 0.24 mm^2^ areas for astrocytomas, *IDH*-mutant and with a mitosis cell count ≥9 per 10 consecutive 0.24 mm^2^ areas for oligodendrogliomas, *IDH*-mutant and 1p19q-codeleted.^36,37^ The attempts to postpone standard oncological treatment in grade 3 glioma *IDH*-mutant patients following maximal safe resection aim to preserve neurocognitive functioning and health-related quality of life without negatively impacting survival.^31^ The present results support such a conservative approach whenever a large resection is obtained. The historical NOA-04 phase III trial demonstrated no survival difference between early radiotherapy and chemotherapy alone in anaplastic gliomas diagnosed between 1999 and 2005.^38,39^ The ongoing IWOT trial (NCT03763422) compares the watch-and-wait approach (treatment as per local practice) with the early treatment (radiotherapy and chemotherapy) in grade 2 and 3 astrocytomas, *IDH*-mutant without a need for immediate postoperative treatment. It will provide useful insights regarding the benefit-to-risk ratio of the watch-and-wait approach and early oncological treatment regarding survival and long-term toxicity.

Our experience supports the feasibility of a watch-and-wait approach in selected patients following maximal safe resection. This supports the transposition of a maximal safe resection, which is the surgical standard of care for *IDH*-mutant diffuse low-grade gliomas,^6,7^ to grade 3 glioma, *IDH*-mutant patients. This powerful technique shall improve the benefit-to-risk of the surgery. In these patients, an additional benefit of achieving large and safe resection will be to postpone adjuvant oncological treatments, reducing the occurrence of long-term radiotherapy-induced side effects and keeping radiotherapy and chemotherapy as powerful treatment options for future progressions. This is a topical question in the context of the near future positioning of *IDH1/2* inhibitors for the treatment of 3-grade gliomas, *IDH*-mutant.

### Generalizability

The strengths of this study lie in (1) the homogeneous population of adult-type grade 3 glioma, *IDH*-mutant patients who underwent the same awake procedure; (2) the standardized data collection and the absence of lost to follow-up allowed for a reliable evaluation of the effects of watch-and-wait approach compared to standard oncological treatment; (3) the comprehensive histo-molecular diagnosis according to the current 2021 WHO classification based on large resections with a low risk of under-sampling; (4) the case-matched analysis to control for selection biases between surgery and oncological groups, including histo-molecular diagnosis, extent of resection, tumor volume, preoperative KPS score, tumor location, and age.

This cohort reflects the outcomes of grade 3 glioma, *IDH*-mutant patients who underwent a watch-and-wait approach following radical resection and could help envision such an approach in selected patients. The clinical relevance of long-term outcomes of a postoperative watch-and-wait approach in these patients remains to be confirmed by further investigations within the context of prospective large databases.

### Limitations

Limitations arose from the observational, retrospective, monocentric study design, the exploratory design of statistical analyses, the limited number of patients, the lack of a control group to compare and validate the results, and the lack of an external validation set. The relatively short follow-up (about 23% of tumor progression, about 15% of death) rendered the survival analyses immature and precluded from definitively assessing the impact of the watch-and-wait approach following radical resection on survivals. Since we did not perform in-depth sequencing and molecular profiling, including the search for the CDKN2A homozygous deletion in grade 3 oligodendrogliomas, *IDH*-mutant, and 1p19q-codeleted, we cannot exclude a molecular imbalance between the surgery and the oncological groups.^40^ By focusing on newly diagnosed grade 3 gliomas, *IDH*-mutant amenable to a large function-based resection, the present results cannot be extrapolated to other diffuse gliomas, including grade 4 astrocytomas, *IDH*-mutant, to biopsied cases, and to high-risk patients. The case-matched analysis encompassed the main prognostic factors but did not include all parameters influencing the clinical decision-making, including corpus callosum involvement, hyperperfusion, or the presence of focal neurological deficit. Therefore, these findings should be interpreted with consideration of these limitations.

## Conclusion

A watch-and-wait approach following radical resection appears to be feasible in highly selected grade 3 astrocytomas, *IDH*-mutant and oligodendrogliomas, *IDH*-mutant and 1p19q-codeleted without impairing survival. Such an approach aims at reducing early radiotherapy with the possible benefit of postponing its long-term side effects to preserve neurocognitive functioning and health-related quality of life. This has to be envisioned in selected patients in an individualized functional neuro-oncology approach and integrated together with the introduction of *IDH1/2* inhibitors. This selected patient-based approach remains to be confirmed by further investigations.

## Supplementary Material

vdae189_suppl_Supplementary_Figures_S1

vdae189_suppl_Supplementary_Figures_S2

vdae189_suppl_Supplementary_Tables_S1-S2

## Data Availability

All datasets analyzed in this study are available from the corresponding author on reasonable request.
