# Supplementary figures and images for: Watch-and-wait approach versus adjuvant treatment after radical awake resection in selected adult-type grade 3 gliomas, isocitrate dehydrogenase mutant: A case-matched cohort
Source: Neurooncol Adv. 2024 Nov 18;6(1):vdae189. doi: 10.1093/noajnl/vdae189 (PMC11606645; doi:10.1093/noajnl/vdae189)

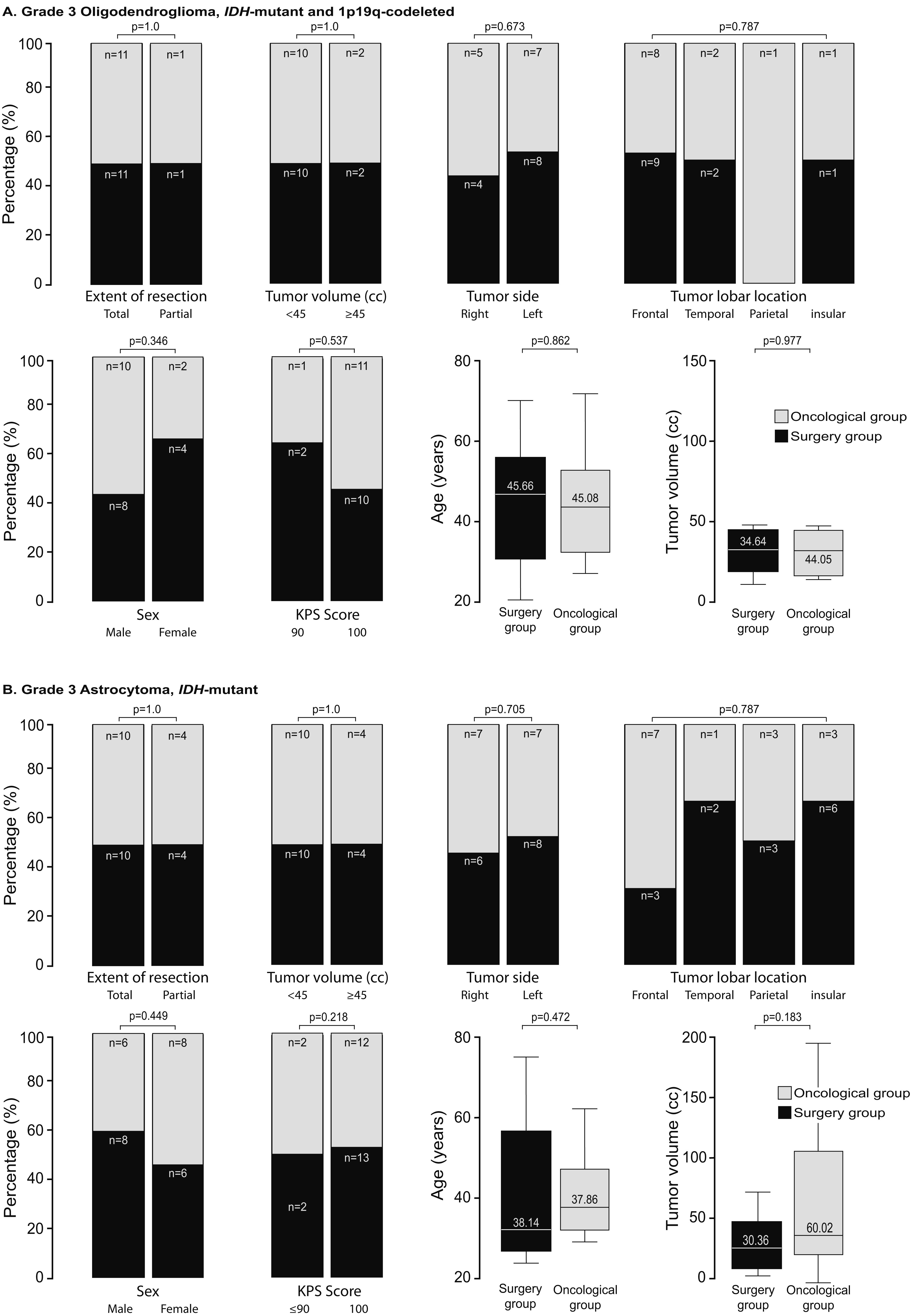

Supplement: vdae189_suppl_Supplementary_Figures_S1 [file vdae189_suppl_Supplementary_Figures_S1.tif]

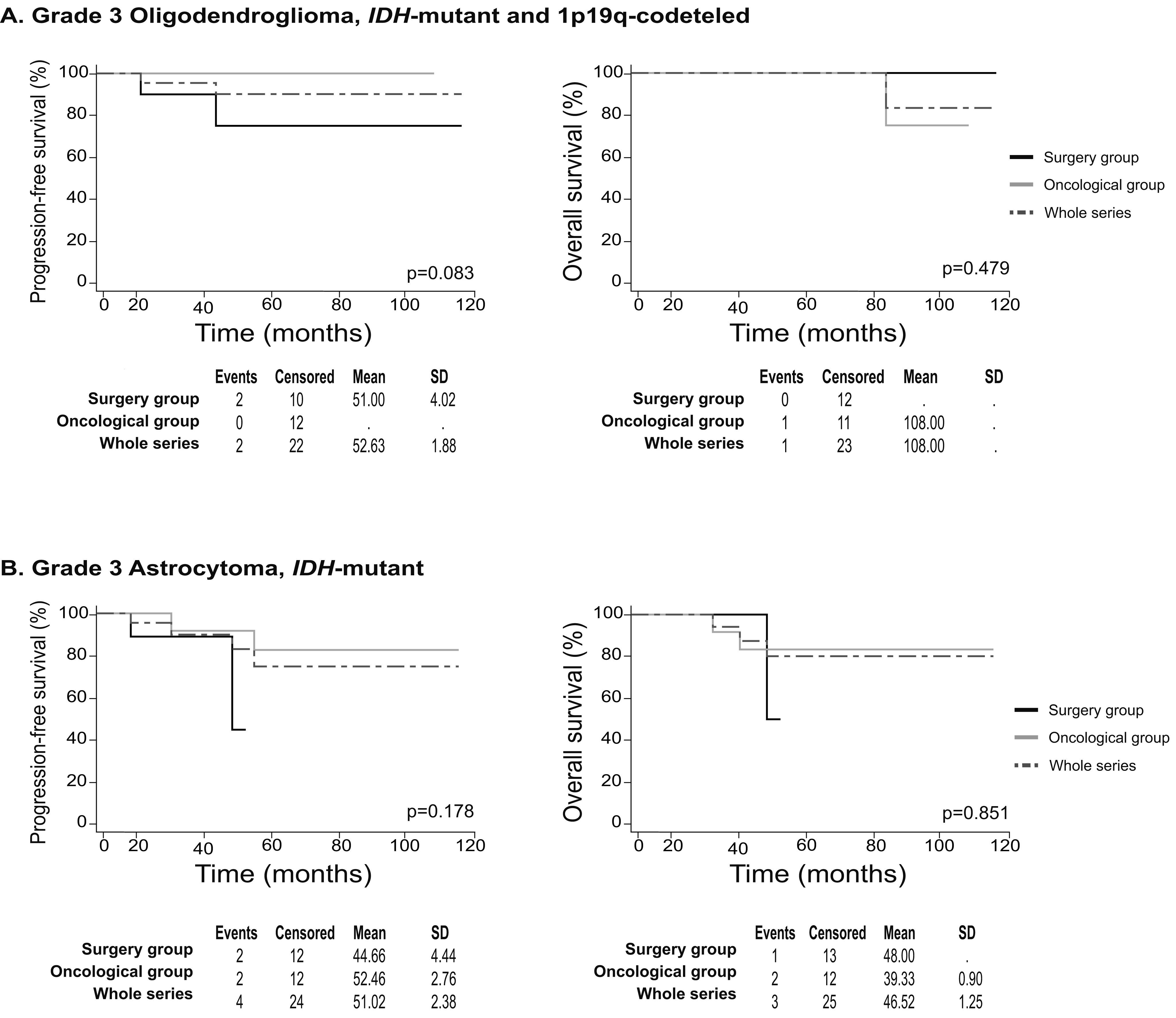

Supplement: vdae189_suppl_Supplementary_Figures_S2 [file vdae189_suppl_Supplementary_Figures_S2.tif]
