# Supplementary material for: Watch-and-wait approach versus adjuvant treatment after radical awake resection in selected adult-type grade 3 gliomas, isocitrate dehydrogenase mutant: A case-matched cohort
Source: Neurooncol Adv. 2024 Nov 18;6(1):vdae189. doi: 10.1093/noajnl/vdae189 (PMC11606645; doi:10.1093/noajnl/vdae189)
Supplement: vdae189_suppl_Supplementary_Tables_S1-S2 [file vdae189_suppl_Supplementary_Tables_S1-S2.docx]

**Supplementary Data**

**Supplementary Table 1.** Surgery and Oncological groups paired by matching criteria.

**Supplementary Table 2.** Characteristics of patients of the surgery group (n=26).

**Supplementary Figure 1.** Detailed comparison of matching criteria in the case-control sampling of grade 3 oligodendrogliomas, *IDH*-mutant and 1p19q-codeleted (A), and of grade 3 astrocytoma, *IDH*-mutant (B).

**Supplementary Figure 2.** Progression-Free Survival and Overall survival after case matching in grade 3 oligodendrogliomas, *IDH*-mutant and 1p19q-codeleted (top row) and in grade 3 astrocytomas, *IDH*-mutant (bottom row).

**Supplementary Table 1. Surgery and Oncological groups paired by matching criteria.**

|  | Grade 3 Oligodendroglioma, *IDH*-mutant and 1p19q-codeleted | | | | | | | | | | | | | | |
| --- | --- | --- | --- | --- | --- | --- | --- | --- | --- | --- | --- | --- | --- | --- | --- |
|  | ***Surgery group*** | | | | | | |  | ***Oncological group*** | | | | | | |
|  | **Sex** | **Age** | **KPS score** | **Side** | **Main location** | **Volume (cm^3^)** | **EOR** |  | **Sex** | **Age** | **KPS score** | **Side** | **Main location** | **Volume (cm^3^)** | **EOR** |
| 1 | F | 62 | 100 | L | Frontal | 108.3 | Total |  | F | 55 | 100 | L | Frontal | 12.2 | Total |
| 2 | M | 49 | 100 | L | Frontal | 45.1 | Partial |  | M | 57 | 100 | L | Frontal | 80.6 | Partial |
| 3 | M | 59 | 100 | L | Frontal | 29.0 | Total |  | M | 42 | 100 | L | Frontal | 40.5 | Total |
| 4 | F | 54 | 90 | R | Frontal | 22.2 | Total |  | F | 45 | 90 | R | Frontal | 14.,8 | Total |
| 5 | M | 29 | 100 | L | Frontal | 9.5 | Total |  | M | 28 | 100 | L | Frontal | 18.6 | Total |
| 6 | M | 72 | 100 | L | Insular | 15.0 | Total |  | M | 60 | 100 | L | Insular | 49.7 | Total |
| 7 | M | 41 | 90 | R | Temporal | 39.5 | Total |  | M | 47 | 100 | R | Temporal | 12.4 | Total |
| 8 | F | 21 | 100 | R | Frontal | 29.0 | Total |  | M | 56 | 100 | R | Frontal | 32.4 | Total |
| 9 | M | 48 | 100 | R | Frontal | 30.6 | Total |  | M | 30 | 100 | R | Frontal | 27.4 | Total |
| 10 | F | 25 | 100 | L | Frontal | 16.5 | Total |  | M | 21 | 100 | L | Frontal | 51.7 | Total |
| 11 | M | 48 | 100 | L | Temporal | 104.1 | Partial |  | M | 51 | 100 | L | Temporal | 128.1 | Partial |
| 12 | M | 40 | 100 | L | Frontal | 27.4 | Total |  | M | 36 | 100 | L | Frontal | 14.2 | Total |
|  | | | | | | | | | | | | | | | |
|  | **Grade 3 Astrocytoma, *IDH*-mutant** | | | | | | | | | | | | | | |
|  | ***Surgery group*** | | | | | | |  | ***Oncological group*** | | | | | | |
|  | **Sex** | **Age** | **KPS score** | **Side** | **Main location** | **Volume (cm^3^)** | **EOR** |  | **Sex** | **Age** | **KPS score** | **Side** | **Main location** | **Volume (cm^3^)** | **EOR** |
| 1 | M | 70 | 80 | L | Insular | 72.0 | Partial |  | F | 50 | 100 | L | Insular | 132.8 | Partial |
| 2 | F | 23 | 100 | R | Parietal | 5.1 | Partial |  | F | 35 | 100 | R | Parietal | 15.6 | Partial |
| 3 | F | 26 | 100 | R | Frontal | 64.0 | Total |  | F | 28 | 90 | R | Frontal | 115.3 | Total |
| 4 | F | 30 | 100 | L | Insular | 28.3 | Total |  | M | 29 | 100 | L | Frontal | 21.6 | Total |
| 5 | M | 31 | 100 | L | Temporal | 17.0 | Total |  | F | 36 | 100 | L | Temporal | 44.9 | Total |
| 6 | M | 23 | 100 | L | Temporal | 37.7 | Total |  | M | 31 | 100 | L | Parietal | 43.7 | Total |
| 7 | M | 60 | 100 | L | Insular | 51.2 | Partial |  | F | 42 | 100 | L | Insular | 104.3 | Partial |
| 8 | M | 25 | 100 | L | Insular | 43.2 | Total |  | M | 28 | 100 | L | Frontal | 18.3 | Total |
| 9 | M | 56 | 100 | L | Insular | 9.8 | Partial |  | F | 58 | 100 | R | Insular | 25.5 | Partial |
| 10 | M | 29 | 100 | R | Frontal | 15.8 | Total |  | F | 32 | 90 | R | Frontal | 41.3 | Total |
| 11 | M | 33 | 100 | L | Insular | 7.4 | Total |  | M | 34 | 100 | L | Frontal | 11.4 | Total |
| 12 | F | 52 | 100 | R | Parietal | 22.1 | Total |  | M | 36 | 100 | R | Parietal | 26.5 | Total |
| 13 | F | 47 | 100 | R | Parietal | 3.5 | Total |  | M | 47 | 100 | R | Frontal | 32.0 | Total |
| 14 | F | 29 | 100 | R | Frontal | 47.9 | Total |  | F | 44 | 100 | R | Frontal | 207.1 | Total |

**EOR**: Extent of resection; **KPS**: Karnofsky Performance Status

**Supplementary Table 2. Characteristics of patients of the surgery group (n=26).**

| **N°** | **Sex** | **Age (years)** | **Presenting**  **symptom** | **KPS score** | **Tumour**  **location** | **Tumour**  **volume (cm^3^)** | **Extent of**  **resection** | **Diagnosis** | **Planned adjuvant**  **treatment** | **Reason for no adjuvant**  **treatment** | **Tumour**  **progression** | **Time interval (months) and treatment** | **Death** | **Follow-up since surgery (months)** |
| --- | --- | --- | --- | --- | --- | --- | --- | --- | --- | --- | --- | --- | --- | --- |
| 1 | F | 62 | Seizures | 100 | L frontal | 108.3 | Total | Oligo | RT plus adjuvant PCV | Patient's decision | No |  | No | 152.0 |
| 2 | M | 49 | Seizures | 100 | L frontal | 45.1 | Partial | Oligo | RT plus adjuvant PCV | Patient's decision | Yes | 54.0 (RT, no histomolecular diagnosis) | No | 141.0 |
| 3 | M | 59 | Seizures | 100 | L frontal | 29.0 | Total | Oligo | RT plus adjuvant PCV | Patient's decision | No |  | No | 82.0 |
| 4 | F | 54 | Seizures | 90 | R frontal | 22.2 | Total | Oligo | RT plus adjuvant PCV | Neurosurgeon's proposal | No |  | No | 67.0 |
| 5 | M | 29 | Seizures | 100 | L frontal | 9.5 | Total | Oligo | RT plus adjuvant PCV | Patient's decision | No |  | No | 66.5 |
| 6 | F | 72 | Deficit | 100 | L insular | 15.0 | Total | Oligo | RT plus adjuvant PCV | Neurosurgeon's proposal | No |  | No | 62.5 |
| 7 | F | 23 | Headaches | 100 | L parietal | 5.1 | Partial | Astro | Wait and watch |  | No |  | No | 52.0 |
| 8 | M | 41 | Incidental | 90 | R temporal | 39.5 | Total | Oligo | RT plus adjuvant PCV | Neurosurgeon's proposal | No |  | No | 49.0 |
| 9 | M | 70 | Seizures | 80 | L insular | 72.0 | Partial | Astro | RT plus adjuvant TMZ | Poor overall condition | Yes | 48.0 (none, no histomolecular diagnosis) | Yes | 48.0 |
| 10 | F | 21 | Incidental | 100 | R frontal | 29.0 | Total | Oligo | RT plus adjuvant PCV | Neurosurgeon's proposal | Yes | 24.0 (PCV, Oligo grade 3) | No | 44.0 |
| 11 | M | 56 | Seizures | 100 | L insular | 9.8 | Partial | Astro | Wait and watch |  | No |  | No | 43.0 |
| 12 | F | 26 | Incidental | 100 | R frontal | 64.0 | Total | Astro | RT plus adjuvant TMZ | Neurosurgeon's proposal | No |  | No | 40.0 |
| 13 | F | 25 | Seizures | 100 | L frontal | 16.5 | Total | Oligo | RT plus adjuvant PCV | Neurosurgeon's proposal | No |  | No | 36.0 |
| 14 | F | 30 | Seizures | 100 | L insular | 28.3 | Total | Astro | RT plus adjuvant TMZ | Neurosurgeon's proposal | No |  | No | 35.0 |
| 15 | M | 31 | Incidental | 100 | L temporal | 17.0 | Total | Astro | Wait and watch |  | No |  | No | 35.0 |
| 16 | M | 60 | Seizures | 100 | L insular | 51.2 | Partial | Astro | RT plus adjuvant TMZ | Neurosurgeon's proposal | No |  | No | 26.0 |
| 17 | M | 23 | Seizures | 100 | L temporal | 37.7 | Total | Astro | Wait and watch |  | No |  | No | 26.0 |
| 18 | M | 25 | Seizures | 100 | L insular | 43.2 | Total | Astro | Wait and watch |  | Yes | 18.0 (surgery, Astro grade 3) | No | 24.0 |
| 19 | M | 48 | Seizures | 100 | L temporal | 43.6 | Total | Oligo | Wait and watch |  | No |  | No | 22.0 |
| 20 | M | 29 | Seizures | 100 | R frontal | 15.8 | Total | Astro | Wait and watch |  | No |  | No | 18.0 |
| 21 | M | 33 | Incidental | 100 | L insular | 7.4 | Total | Astro | RT plus adjuvant TMZ | Neurosurgeon's proposal | No |  | No | 13.0 |
| 22 | F | 52 | Seizures | 100 | R parietal | 22.1 | Total | Astro | Wait and watch |  | No |  | No | 13.0 |
| 23 | F | 47 | Incidental | 100 | R parietal | 3.5 | Total | Astro | Wait and watch |  | No |  | No | 12.8 |
| 24 | M | 48 | Incidental | 100 | R frontal | 30.6 | Total | Oligo | RT plus adjuvant PCV | Neurosurgeon's proposal | No |  | No | 12.5 |
| 25 | M | 40 | Seizures | 100 | L frontal | 27.4 | Total | Oligo | RT plus adjuvant TMZ | Neurosurgeon's proposal | No |  | No | 12.0 |
| 26 | F | 29 | Seizures | 100 | R frontal | 47.9 | Total | Astro | Wait and watch |  | No |  | No | 12.0 |

**Astro**: astrocytoma; **F**: female; **KPS**: Karnofsky Performance Score; **L**: left; **M**: male; **Oligo**: oligodendroglioma; **PCV**: Procarbazine, CCNU, and Vincristine; **R**: right; **RT**: radiotherapy; **TMZ**: temozolomide.
